# Absence of TLR4 Reduces Neurovascular Unit and Secondary Inflammatory Process after Traumatic Brain Injury in Mice

**DOI:** 10.1371/journal.pone.0057208

**Published:** 2013-03-28

**Authors:** Akbar Ahmad, Rosalia Crupi, Michela Campolo, Tiziana Genovese, Emanuela Esposito, Salvatore Cuzzocrea

**Affiliations:** 1 Department of Clinical and Experimental Medicine and Pharmacology, School of Medicine, Messina, Italy; 2 University of Manchester, Manchester, United Kingdom; Georgia Health Sciences University, United States of America

## Abstract

**Background:**

Traumatic brain injury (TBI) initiates a neuroinflammatory cascade that contributes to neuronal damage and behavioral impairment. Toll-like receptors (TLRs) are signaling receptors in the innate immune system, although emerging evidence indicates their role in brain injury. We have therefore investigated the role played by TLR4 signaling pathway in the development of mechanisms of secondary inflammatory process in traumatic brain injury (TBI) differ in mice that lack a functional TLR4 signaling pathway.

**Methods/Principal Findings:**

Controlled cortical impact injury was performed on TLR4 knockout (KO) mice (C57BL/10ScNJ) and wild-type (WT) mice (C57BL/10ScNJ). TBI outcome was evaluated by determination of infarct volume and assessment of neurological scores. Brains were collected at 24 h after TBI. When compared to WT mice, TLR4 KO mice had lower infarct volumes and better outcomes in neurological and behavioral tests (evaluated by EBST and rotarod test). Mice that lacked TLR4 had minor expression of TBI-induced GFAP, Chymase, Tryptase, IL-1β, iNOS, PARP and Nitrotyrosine mediators implicated in brain damage. The translocation of expression of p-JNK, IκB-α and NF-κB pathway were also lower in brains from TLR4 KO mice. When compared to WT mice, resulted in significant augmentation of all the above described parameters. In addition, apoptosis levels in TLR4 KO mice had minor expression of Bax while on the contrary with Bcl-2.

**Conclusions/Significance:**

Our results clearly demonstrated that absence of TLR4 reduces the development of neuroinflammation, tissues injury events associated with brain trauma and may play a neuroprotective role in TBI in mice.

## Introduction

Traumatic brain injury (TBI) represents a major public health problem, with >1.7 million new cases annually in the United States alone, accounting for 60% of all emergency department admissions and 50% of all trauma deaths [1]. Traumatic brain injury causes cell death and neurologic dysfunction through both direct physical disruption of tissue (primary injury), as well as through delayed and potentially reversible molecular and cellular pathophysiological mechanisms, which cause progressive white and gray matter damage (secondary injury) [2], [3]. Such delayed injury begins within seconds to minutes after the insult and may continue for days, weeks, or potentially months to years [2] and may be responsible for a significant component of neurodegeneration and neurologic impairment after TBI [4]. Some of the more important secondary injury mechanisms involve intrinsic neuronal cell death pathways, microglial activation. As the main cause of aggravation of brain injury, inflammatory cascade reactions cause specific activities. There are inflammatory cells aggregations, up-regulation of cytokines expression of intercellular adhesion molecules during brain injury, and, what is more, oriented anti-inflammatory therapy has displayed evident neuroprotective effects on animal models. The inflammatory response is usually associated with the activation of innate immunity, specifically with Toll-like receptors (TLRs), the key host molecules in the regulation of the immune response during infections and CNS damage.

TLRs are a family of at least 11 proteins that function as key mediators of innate immunity [5]. TLRs are pattern-recognition receptors that enable the recognition of conserved structural motifs in a wide array of pathogens. It was shown that the mammalian TLR4 protein had been adapted primarily to recognize lipopolysaccharide (LPS), a major cell wall component of Gram-negative bacteria [6]. Activation of TLR4 triggers the downstream stimulation of nuclear factor-κB (NFκB) inducing kinase, a member of the mitogen-activated protein kinase family, and the pathway leading to activation of IκB kinases (IKKα and IKKβ), which in turn phosphorylate IκB and promote its degradation [7]. IκB degradation leads to the release of NFκB subunit enabling NFκB to translocate to the nucleus and initiate gene transcription [8] and the induction of genes that encode inflammation-associated molecules and cytokines [9], [10]. TLR4 signaling also leads to the activation of ERK and JNK signaling pathways [8]. Most TLRs are expressed in the CNS, mainly in glial cells [11]. Recent evidence demonstrates that these receptors respond to pathogens and host tissue injury [12], [13] and they not only play a role in the innate immunity in response to infections but also participate in CNS neurodegeneration and neural injury [14], [15].

Therefore, by considering the critical role of TLR4 in the neuroinflammation and cerebral ischemia, the present study aims to establish the potential role of TLR4 in the TBI. Here we show that TLR4 is critical for brain injury mediated inflammatory signaling in astrocytes, since the knockdown of TLR4, by using cells from TLR4-deficient mice (TLR4 KO), abolished not only the activation of microtubule-associated protein kinase (MAPK) such as JNK and NFκB signaling pathways but also the production of inflammatory mediators by astrocytes. Our results also demonstrate, for the first time, that although TBI causes significant microglial and astroglial activation (GFAP), Chymase, Tryptase and increases IL-1β, inducible nitric oxide synthase (iNOS), PARP, Nitrotyrosine expression in the cerebral cortex of wild-type (WT) mice compare to TLR4 KO mice. Moreover, TLR4 KO mice having improvements in tissues histology shown by reduction of lesion size and apoptosis level reduced Bax expression while on the contrary Bcl-2 expression. In addition, we demonstrated that the absence of TLR4 in TLR4 KO mice improved neurobehavioral functions as evaluated by motor deficits when compared with WT mice. These results support the pivotal role of the TLR4 response in the neuroinflammation, tissues injury events associated with brain trauma.

## Results

### Neurological deficits after Brain injury

**Figure 1:**
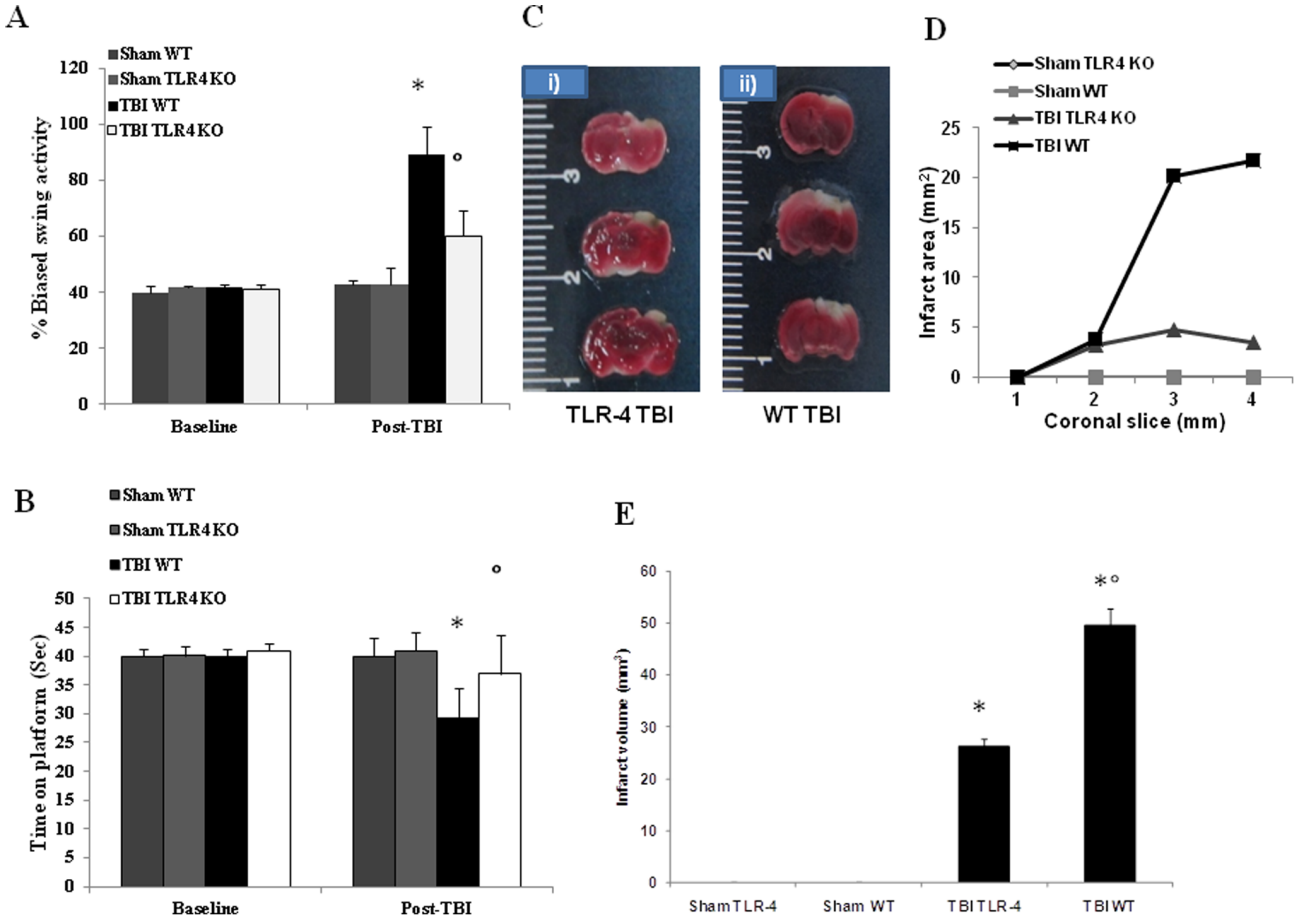
Effect of absence of TLR4 on brain infarctions following TBI improves behavioral function. Post-injury following TBI improved motor function evaluated by EBST (A) and rotarod test (B), after 24 h. TLR4 KO mice showed significant impairments in motor deficits as revealed by significantly biased swing activity (A) and shortened time to stay on rotating rod (B) compare to WT mice. Representative TTC stained brain section (#3 out of the six consecutive sections from cranial to caudate region) corresponding to largest infraction from each group (C). Brain sections (2 mm thick) were stained with TTC at 24 h after TBI to shows significant difference in TLR4 KO and WT mice in terms of area (D) and volume (E) of infarctions. The figures are representative of at least three experiments performed on different experimental days. Each data are expressed as Mean ± SEM from N = 10 Mice for each group. A *p*-value of less than 0.05 was considered significant.*p<0.01 vs. Sham, °*p*<0.01 vs *WT-TBI*.

Neurological deficit was evaluated using two different tests. We used the EBST, rotarod test, which is reportedly the most sensitive vestibulomotor measure. In both behavioral tests, we found TBI-TLR4 KO mice gradually but significantly improved latency and neurological deficits compared to TBI-WT mice (Fig. 1A, B).

### Infarct Outcome in TLR4-Deficient Mice after TBI

Brain edema indicates pathology associated with endothelial cell activation and endothelial dysfunction. To evaluate the effect of absence of TLR4 on brain edema and infarctions in the TBI brain, we performed TTC staining (Fig. 1C, i and ii). At 24 h after TBI, TLR4 KO mice had significantly smaller infarct area (Fig. 1D) and volume (Fig. 1E) compare to WT mice.

### Severity of Brain trauma and MCs infiltration in TLR4-Deficient Mice after TBI

**Figure 2:**
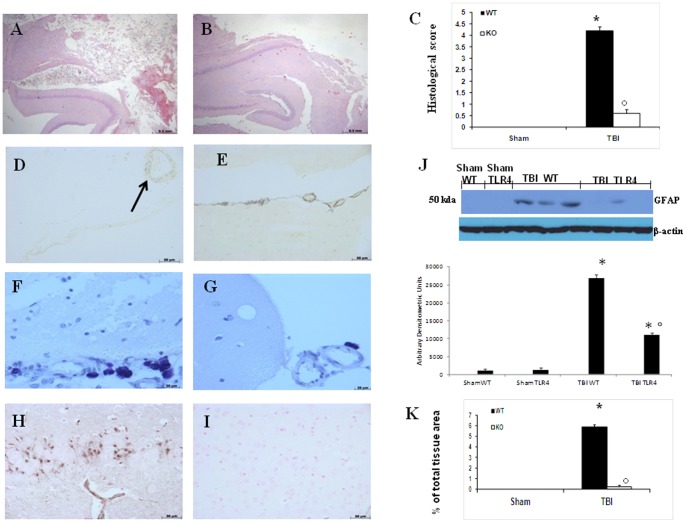
Severity of Brain trauma on histological alterations and MCs infiltration in TLR4-Deficient Mice after TBI. Significant damage to the brain tissue in WT mice was apparent, as demonstrated by the presence of edema as well as alteration of the frame and a significant alteration of reticular and nervous fibers (A). A significant protection from TBI-induced damage and reduced alteration of reticular and nervous fibers were observed in the tissue samples from TLR4 KO mice (B). The low-magnification images of brain after TBI show tissue disorganization, white matter alteration, and inflammation in the perilesional area at 24 h after injury (D). A significant protection from the TBI was evident in the tissue collected from TLR4 KO mice (E). The slides stained with acidified Toluidine blue were also shown to have dark-lilac blue granules, which identify the cells as mast cells. Many of the mast cells are arranged separately in concentric rings around small blood-vessels. Different gradients of staining intensity were evident in the granulated and degranulated mast cells. In the brain tissues collected from WT mice at 24 h after TBI there is the presence of MCs (F) mainly localized in the perivascular area. On the contrary, TLR4 KO mice reduced MC infiltration in the brain tissue after TBI (G). A significant increase in GFAP expression, assayed by western blot analysis, was detected in the tissue from (J). TLR4 KO mice show reduced GFAP expression (J) in the brain. Moreover, in tissue sections obtained from WT mice as compared with TLR4 KO mice demonstrate positive staining for GFAP mainly localized in activated astrocytes (H). The figures are representative of at least three experiments performed on different experimental days. Each data are expressed as Mean ± SEM from N = 10 Mice for each group. *p<0.01 vs. Sham, °*p*<0.01 vs *WT-TBI*.

In order to better study the severity of the trauma at the level of the perilesional area, the brain tissues were collected at 24 h after injury and stained by Haematoxylin/Eosin (H&E). The histological evaluations showed prominent and thickened blood vessels, ischemic changes and gliosis in the brain parenchyma (Fig. 2A and 2B see histological score 2C). Infiltration of the parenchyma by numerous macrophages, scattered lymphocytes, and occasional neutrophils was also evident (Fig. 2A and 2B). In this study we found that the TLR4 KO mice had significantly reduced the brain damage (Fig. 2B) at 24 h after trauma. The low-magnification images of brain after TBI show tissue disorganization, white matter alteration, and inflammation in the perilesional area at 24 h after injury (2D). A significant protection from the TBI was evident in the tissue collected from TLR4 KO mice (2E). The histological pattern of TBI described above appeared to be correlated with cellular changes into the brain. In particular, a presence of MCs was observed in the brain tissues collected at 24 h after TBI (Fig. 2F and 2G) mainly localized in the perivascular area. Cells showing to have metachromatic granules when stained with acidified Toluidine blue were identified as mast cells. On the contrary, significant less MCs density and degranulation were observed in the brain tissues after TBI collected from TLR4 KO mice (Fig. 2G) compare to WT mice. No granules were found in the brain tissues from both sham-operated TLR4 KO and WT mice (data not shown).

### Effect of absence of TLR4 on GFAP after TBI

At 24 h after TBI, GFAP was measured by immunohistochemical and western blot analysis in the brain sections. GFAP has been implicated in the pathogenesis of TBI. Accordingly, we used an immunohistochemical approach to assess the presence of GFAP, astrocytic marker. As shown in Fig. 2 (Fig. 2H, see densitometry analysis K), there were positive staining for GFAP localized in the brain tissues from WT mice subjected to TBI compare to TLR4 KO mice subjected to TBI (Fig. 2 I, see densitometry analysis K). No staining was found in the brain tissues from both sham-operated TLR4 KO and WT mice (data not shown). We also evaluated GFAP expression by western blot analysis to confirm the cellular mechanisms in TBI. The level of GFAP in cytoplasmic fraction was decreased after TBI in TLR4 KO compare to WT mice (Fig. 2 J, see densitometry analysis). There were no significant differences among sham-operated WT and TLR4 KO.

### Effect of absence of TLR4 on chymase and tryptase expression after TBI

**Figure 3:**
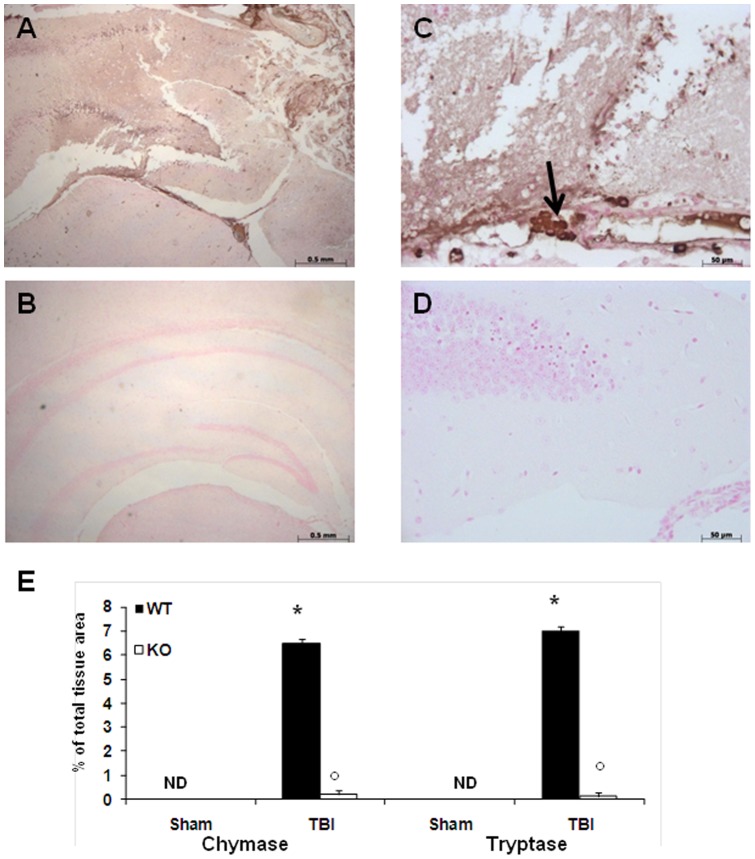
Effect of absence of TLR4 on chymase and tryptase expression after TBI. A substantial increase in serine peptidases chymase and tryptase expression was found mainly localized in MCs in the brain tissues collected from WT mice at 24 h after TBI (A), (C). Chymase and tryptase levels were attenuated in TLR4 KO mice (B), (D). This figure is representative of at least three experiments performed on different experimental days. Each data are expressed as Mean ± SEM from N = 10 Mice for each group. *p<0.01 vs. Sham, °*p*<0.01 vs *WT-TBI*.

In order to test whether absence of TLR4 may modulate and direct the inflammatory response through the regulation of the serine peptidases, we analyzed by immunohistochemistry the brain expression of chymase and tryptase. There was no staining for chymase and tryptase in the brain tissues obtained from the sham-operated WT and TLR4 KO mice (data not shown). A substantial increase in chymase and tryptase expression was found mainly localized in MCs in brain tissues collected at 24 h after TBI from WT mice (Figs. 3A, C, see densitometry analysis E). Expression of chymase and tryptase were attenuated in the brain from TLR4 KO mice. (Figs. 3B, D, see densitometry analysis E).

### Effect of absence of TLR4 on IL1-β and iNOS inhibition after TBI

**Figure 4:**
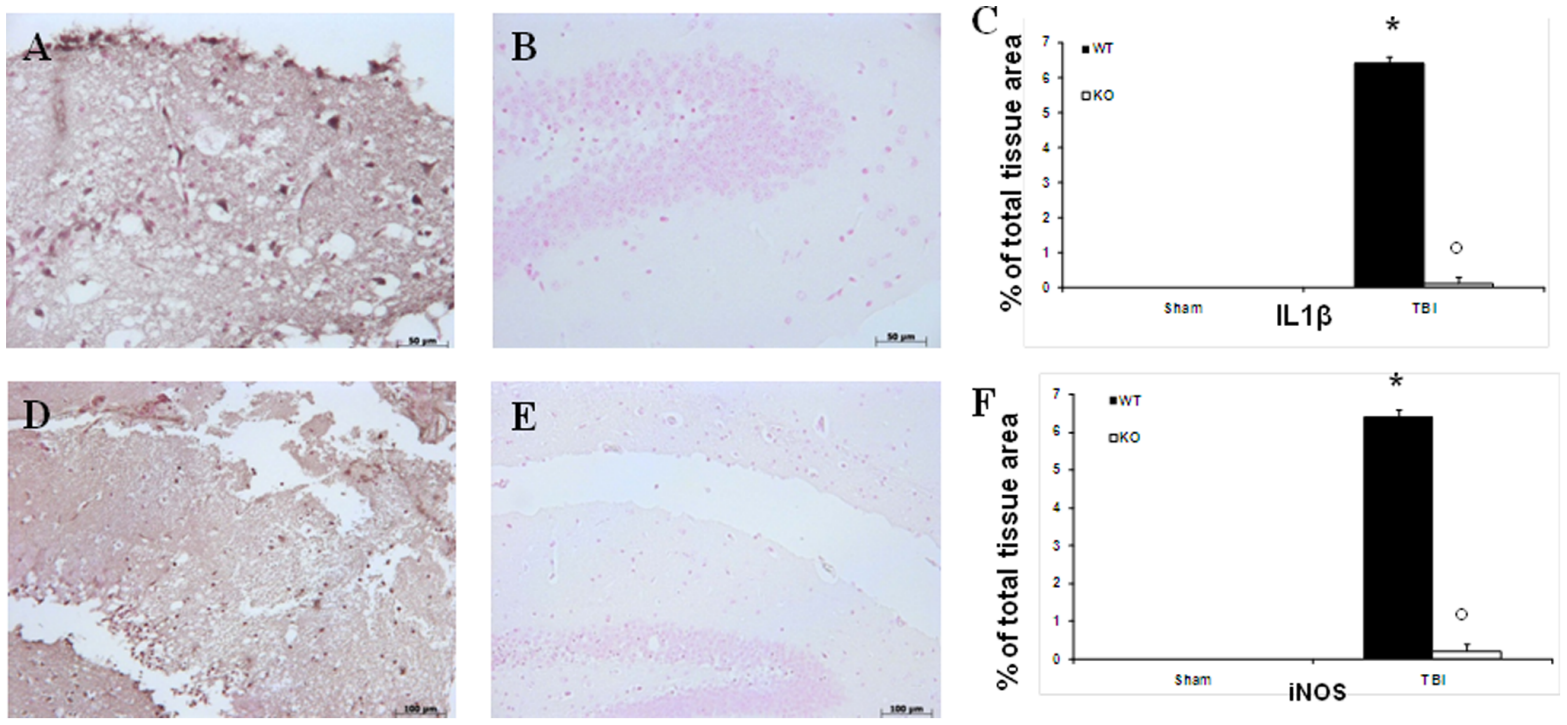
Effect of absence of TLR4 on IL-1β and inducible nitric oxide synthase after TBI. For immunohistological staining for IL-1β and iNOS. Brain sections obtained from TBI-induced WT mice exhibited a positive staining for IL-1β and iNOS (A, D). Absence of TLR4 significantly reduced staining for IL-1β and iNOS (B, E). The figures are representative of at least three experiments performed on different experimental days. Each data are expressed as Mean ± SEM from N = 10 Mice for each group. A *p*-value of less than 0.05 was considered significant. *p<0.01 vs. Sham, °*p*<0.01 vs *WT-TBI*.

We evaluated levels of IL1-β after TBI, samples of brain tissue taken at 24 h after TBI were processed for immunohistological staining for IL1-β. Brain sections from sham-operated WT and TLR4 KO mice did not stain for IL1-β (data not shown) whereas brain sections obtained from TBI-WT mice exhibited a positive staining for IL1-β (Fig. 4A, see densitometry analysis C) whereas, expression of IL1-β was attenuated in the brain from TLR4 KO mice subjected to TBI (Fig. 4B, see densitometry analysis C). Moreover, Samples of brain tissue taken at 24 h after TBI were processed for immunohistological staining for iNOS. Brain sections from sham-operated WT and TLR4 KO mice did not stain for iNOS (data not shown) whereas brain sections obtained from TBI-WT mice exhibited a positive staining for iNOS (Fig. 4D, see densitometry analysis F) whereas, expression of iNOS was attenuated in the brain from TLR4 KO mice subjected to TBI (Fig. 4E, see densitometry analysis F).

### Effect of absence of TLR4 on IκB-α degradation, NF-κB p65 and P-JNK expression in TBI

**Figure 5:**
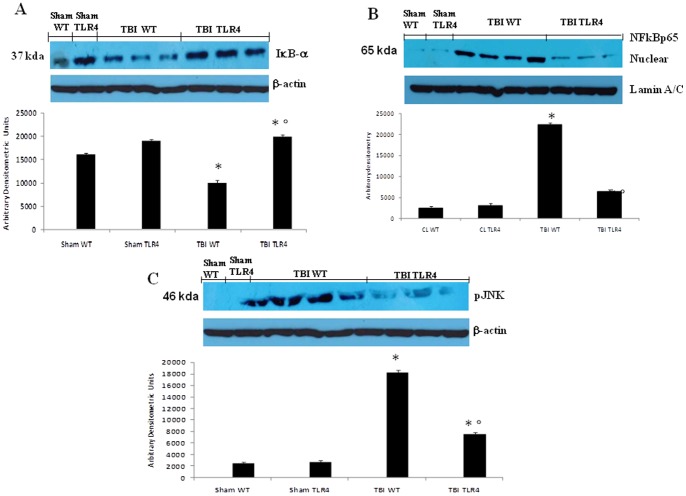
Effect of absence of TLR4 on IκB-α, NF-κB p65 and P-JNK. IκB-α levels were reduced substantially in brain tissues obtained from TBI-operated WT mice (A). TLR4 KO mice prevented TBI-induced IκB-α degradation (A). NF-κB p65 levels in the TBI-induced WT mice brain nuclear fractions were increased significantly compared with the TBI-induced TLR4 KO mice (B). A representative blot of lysates obtained from five animals per group is shown, and densitometry analysis of all animals is reported. A significant increase of p-JNK (C) was observed in brain tissues obtained from TBI treated WT mice compared with the TBI treated TLR4 KO. The figures are representative of at least three experiments performed on different experimental days. Each data are expressed as Mean ± SEM from N = 10 Mice for each group. A *p*-value of less than 0.05 was considered significant. *p<0.01 vs. Sham, °*p*<0.01 vs *WT-TBI*.

We evaluated IκB-α degradation, nuclear NF-κB p65 and P-JNK expression by western blot analysis to investigate the cellular mechanisms whereby TLR4 in the development of TBI. Basal expression of IκB-α was detected in cerebral samples from sham-operated TLR4 KO and WT animals, whereas IκB-α levels were substantially reduced in the brain tissue collected from WT mice compare to TLR4 KO mice at 24 h after TBI (Fig. 5 A). Moreover, NF-κB p65 levels from brain tissue collected from WT mice at 24 h after TBI were also significantly increased compared to the sham-operated WT and TLR KO mice rats (Fig. 5 B).

At 24 h after TBI, the expression of phospho-JNK in brain homogenates was also investigated. A significant increase in phospho-JNK levels were observed in the brain from WT mice compare to TLR4 KO mice subjected to TBI.

### Effect of absence of TLR4 on Nitrotyrosine and PAR formation after TBI

**Figure 6:**
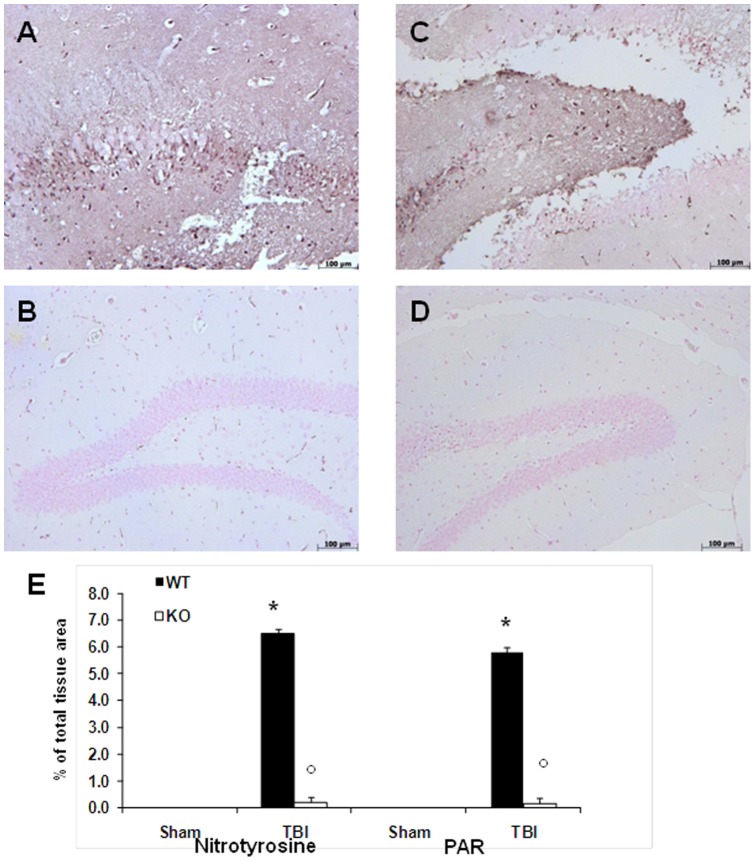
Effect of absence of TLR4 on Nitrotyrosine and PAR formation after TBI. Sections obtained from TBI-induced WT mice demonstrate positive staining for nitrotyrosine (A) mainly localized in inflammatory, in Schwann cells in the white and gray matter. TLR4 KO mice after TBI induction reduced the degree of positive staining for nitrotyrosine (B) in the brain. PAR expression, as an indicator of *in vivo* PARP activation, revealed the occurrence of positive staining localized in nuclei of Schwann cells in the white and gray matter of the brain tissues from WT mice subjected to TBI (C). TBI-induced TLR4 KO mice having reduced the degree of positive staining for PAR (D) in the brain sample. Figures are representative of at least 3 experiments performed on different experimental days. Densitometry analysis of immunocytochemistry photographs for nitrotyrosine and for PAR (E) from brain tissues assessed. The figures are representative of at least three experiments performed on different experimental days. Each data are expressed as Mean ± SEM from N = 10 Mice for each group. A *p*-value of less than 0.05 was considered significant. *p<0.01 vs. Sham, °*p*<0.01 vs *WT-TBI*.

Twenty-four hours after TBI, nitrotyrosine, a specific marker of nitrative stress, was measured by immunohistochemical analysis in the brain sections to determine the localization of various reactive nitrogen species produced during TBI. Brain sections obtained from TBI mediated WT mice exhibited positive staining for nitrotyrosine (Fig. 6 A, see densitometry analysis E). The positive staining was mainly localized in inflammatory cells of the brain tissues. Brain section obtained from TLR4 KO mice subjected to TBI found reduced the degree of positive staining for nitrotyrosine (Fig. 6B, see densitometry analysis E). In addition, at 24 h after TBI, Activation of the enzyme, poly-ADP-ribose polymerase (PARP) has been implicated in the pathogenesis of TBI. Accordingly, we used an immunohistochemical approach to assess the presence of PAR, as an indicator of in vivo PARP activation. There was positive staining for PAR of the brain tissues from WT mice (Fig. 6C, see densitometry analysis E) compare to TLR4 KO mice subjected to TBI.

### Effect of absence of TLR4 on analysis for Bax and Bcl-2

**Figure 7:**
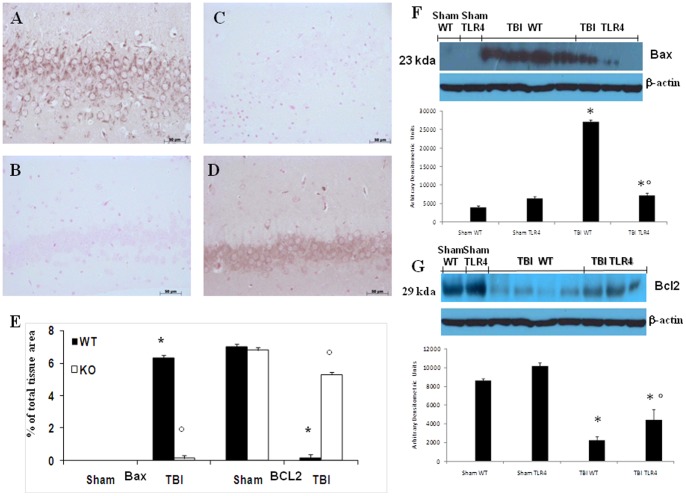
Effects of absence of TLR4 on Bax and Bcl-2 after TBI by western blot and immunohistochemistry analysis. At 24 h, an increase in Bax expression in TBI-induced WT mice as evidenced by Western blot analysis as well as immunohistochemistry (A, F). TLR4 KO mice after TBI induction reduced the degree of positive staining and expression for Bax in the brain (B, F). On the contrary, positive staining for Bcl-2 was observed in the brain tissues from TBI induced TLR4 KO mice (D, G) while the staining was significantly reduced in TBI induced WT mice (C, G). Densitometry analysis of immunohistochemistry photographs for Bax and Bcl-2 (E) from Brain tissues assessed. The figures are representative of at least three experiments performed on different experimental days. Each data are expressed as Mean ± SEM from N = 10 Mice for each group. A *p*-value of less than 0.05 was considered significant. *p<0.01 vs. Sham, °*p*<0.01 vs *WT-TBI*.

Samples of brain tissue taken at 24 h after TBI were processed for western blot technique as well as immunohistological staining for Bax and Bcl-2. Brain sections from sham-operated mice did not stain for Bax (data not shown) whereas brain sections obtained from TBI-WT mice exhibited a positive staining for Bax (Fig. 7A see densitometry analysis E) compare to TBI-TLR4 KO mice (Fig. 7 B see densitometry analysis, E) whereas contrary result with staining for Bcl-2 (Fig. 7 C, D see densitometry analysis, E). Moreover, we also found similar result for Bax and Bcl2 expression by Western blot technique (Fig. 7F, G).

## Discussion

TBI initiates a series of cellular and molecular events, evolving over the following hours and days, causes neuronal apoptosis, inflammation, and reactive gliosis, which contribute to secondary tissue loss, impaired regeneration, and associated functional disabilities [24]–[26]. The CNS was once thought to be an immune-privileged site, but researchers now recognize the role of immunity in the CNS. Microglia are the resident immune cells of the CNS, comprising about 12% of the cells in the brain and spinal cord [27]. It makes sense that innate immune receptors such as TLR4 would be expressed on the immune cells of the nervous system, the microglia. TLR4 is primarily expressed on glia, primarily microglia [28]; however TLR4 expression has been reported on other CNS cells including astrocytes, inflammatory cells, endothelial cells and neurons [29], [30]. In our study, we have revealed the role of TLR4 in adult mice brain after TBI by comparing the result with KO and WT. TLR4-deficient mice have smaller infarct area and less inflammatory response after TBI. We also found that mice (C57BL/10ScNJ) that lack expression of TLR4 present a significantly better brain injury outcome, as shown by a remarkable reduction of infarct volume and a substantial recovery in the neurological deficits induced by TBI compared with mice (C57BL/10ScSn) that express TLR4 normally. The improvement in the neurological function was studied with 2 different assessments: Elevated Biased Swing Test, a simple neurological test and Rotarod test, a behavioral test that widely used. These data demonstrate that TLR4 signaling is involved in brain damage and in inflammation triggered by TBI. This study supports this hypothesis and demonstrates thatTLR4 is critical for the TBI-induced inflammatory signaling in astrocytes, since the knockdown of TLR4 abolished the activation of the MAPK and NFκB pathways as well as the production of inflammatory mediators by astrocytes. TBI promotes glial activation, enhances NFκB-p65 nuclear translocation, and upregulates inflammatory factors in cortices of WT mice, deficiency in the TLR4 function protects against these deleterious events. These results demonstrate the relevance of the TLR4 response in the TBI-induced neuroinflammation and brain damage for the first time. MCs possess several biological mediators that are released from cytoplasmic granules primarily due to stimulus-induced degranulation, including vasoactive amines such as histamine, proteoglycans (mainly heparin and chondroitin sulphate), neutral serine proteases, such as tryptase and chymase, cytokines, astrocytes and growth factors, such as vascular endothelial growth factor, basic fibroblast growth factor, Nerve growth factors [31]–[33]. Activation of both the TLR receptors and glial cells contributes to brain inflammation and neurodegeneration [15], [30], [34], [35]. Most of the TLRs are expressed in microglia and astrocytes [34] and their expression can be altered in pathological states. Astrocytes play important roles in the repairing and scarring processes after brain and spinal cord injury [35]. Indeed, reactive hypertrophic astrogliosis is a marker of neuroinflammation and is also associated with the neurodegenerative disorders [36]–[38]. Our immunohistochemical and westernblot data reveal that TBI induces astroglial activation and astrogliosis in the cerebral cortices of WT mice compare to TLR4 KO mice, as demonstrated by the marked upregulation of GFAP immunoreactivity along with hypertrophic astrocytes, which are events associated with CNS injury [39]–[41].

In absence of pathological conditions, homeostasis of the central nervous system is controlled and well-balanced, and the inflammation significantly contributes to repair of damaged tissues after injury [42], [43]. However, excessive inflammation damages surrounding healthy tissue. Microglia release pro-inflammatory and cytotoxic factors, including IL-1β, IL-6, TNF-α, nitric oxide, reactive oxygen species etc [44]. In this paper, we observed the relationship between the lack of TLR4, which play an important role on secondary events after TBI. It has been well demonstrated that in TBI the expression of proinflammatory cytokines (IL-1β), at the site of injury regulates the precise cellular events after TBI [45]. In the present study, we have clearly demonstrated that, a significant increase of positive immunohistochemical staining for IL-1β in WT-TBI mice in comparison with TLR4 KO mice.

In the search for mechanisms involved in this effect, we found that TBI-induced iNOS expression is significantly lower in brains from TLR4-deficient mice than in those from control mice. We have mentioned that TLRs activate nuclear factor κ-B signaling pathways that produce the transcription of many proinflammatory genes and enzymes such as iNOS [46]. It has been described recently that TLR4 mediates iNOS expression in macrophages in a MyD88-independent (TRIF dependent) manner [47]. Because iNOS mediates cytotoxicity in many cell systems such as the brain injury and ischemic brain [48], [49].

In particular it has been demonstrated that peroxynitrite likely contributes to inflammation through pathways resulting from the chemical modification of cellular proteins and lipids [50]. When NO is produced in the setting of oxidative stress, the highly reactive peroxynitrite is formed, leading to macromolecule damage and the formation of the protein adduct, nitrotyrosine. To confirm the pathological contributions of peroxynitrite to brain inflammation, we have demonstrated here formation of nitrotyrosine in the injured tissue and here we found TLR4-TBI KO mice reduced the immunostaining for nitrotyrosine compare to WT-TBI mice. Nitrotyrosine formation, along with its detection by immunostaining, was initially proposed as a relatively specific marker for the detection of the endogenous formation “footprint” of peroxynitrite. Although increased nitrotyrosine staining is considered, as an indication of “increased nitrosative stress” rather than a specific marker of the peroxynitrite generation. Various studies have demonstrated that PARP activation after single DNA strand breakage induced by ROS plays an important role [51]. In this study we confirm, the increase in PARP formation in the brain tissues from TBI animals as well as that TLR4 KO mice groups attenuates PARP activation. Generation of ROS has been implicated in induction of cell death and inflammation [52]. Moreover, it is well known that Bax, a pro-apoptotic gene, plays an important role in developmental cell death [53] and CNS injury [54]. Similarly, it has been shown that the administration of Bcl-xL fusion protein, (Bcl-2 is the most expressed anti-apoptotic molecule in adult central nervous system) into ischemic brain significantly increased neuronal survival, suggesting that brain injury-induced changes in Bcl-xL contribute considerably to neuronal death [55]. Based on these evidences, we have identified in TBI animals proapoptotic transcriptional changes, including up regulation of proapoptotic Bax and down regulation of antiapoptotic Bcl-2, by westernblot technique and also by Immunohistochemical staining. Our data demonstrate that mice with a targeted deletion of TLR4 gene are less vulnerable to pathologic events in secondary damage associated with TBI compared to WT mice.

In Conclusion, Absence of TLR4 protects against TBI by reducing the exacerbations in the neurovascular unit which are involved in the edema, infraction, cell death, and neurobehavioral deficits in mice following CCI. Considering that the immunity response takes place early after the injury, it would be useful to develop new therapies to inhibit TLR4 signal through the use of neutralizing antibodies or drugs with antagonist characteristics to produce a neuroprotective effect.

## Materials and Methods

### Animals

Adult male C57BL/10ScNJ mice (20–22 g; Charles River; Milan; Italy) with a targeted disruption of the Toll like receptor 4 gene (KO) and littermate wild-type controls (WT) were housed in a controlled environment and provided with standard rodent chow and water. The study was approved by the University of Messina Review Board for the care of animals. Animal care was in compliance with Italian regulations on protection of animals used for experimental and other scientific purposes (D.M.116192) as well as with the EEC regulations (O.J. of E.C. L 358/1 12/18/1986).

### Controlled cortical impact (CCI) model of TBI

Surgical anesthesia was induced by chloral hydrate (400 mg/kg body weight) administered intraperitoneally (i.p,). Following endotracheal intubation, the animals were secured in a stereotaxic frame and ventilated mechanically. A dab of sterile ophthalmic ointment was placed on each eye to compensate for the decrease in lacrimation during anesthesia. Utilizing aseptic techniques, a midline scalp incision was made, and the skin and fascia were reflected to expose the skull. A craniotomy was made in the right hemisphere encompassing bregma and lambda and between the sagittal suture and the coronal ridge with a Micro motor hand piece and drill (UGO Basile S.R.L., Comerio VA, italy). The resulting bone flap was removed, and the craniotomy enlarged further with cranial rongeurs. A cortical contusion was produced on the exposed cortex using a controlled impactor device Impact One^TM^ Stereotaxic impactor for CCI (myNeurolab.com, Richmond). Briefly, the impacting shaft was extended, and the impact tip was centered and lowered over the craniotomy site until it touched the dura mater. Then, the rod was retracted and the impact tip was advanced farther to produce a brain injury of moderate severity for mice (tip diameter, 4 mm; cortical contusion depth, 3 mm; impact velocity, 1.5 m/sec). The impact tip was wiped clean with sterile alcohol after each impact and cleaned/disinfected further with cidex after surgery. Core temperature was maintained at 37±0.5°C with a heating pad during surgery and recorded with a rectal probe. Immediately after injury, the skin incision was closed with nylon sutures, and 2% lidocaine jelly was applied to the lesion site to minimize any possible discomfort.

### Experimental groups

Mice were randomly allocated into the following groups: (i) *TBI C57BL/10ScNJ WT group*. C57BL/10ScNJ WT mice were subjected to TBI (n = 30), (ii) *TBI C57BL/10ScNJ (TLR-4 KO) group*. C57BL/10ScNJ (TLR-4 KO) mice subjected to TBI (n = 30). (iii) *Sham C57BL/10ScNJ WT group*. C57BL/10ScNJ WT mice were subjected to the surgical procedures, as reported above, except that the impact was not given (n = 30). (iv) *Sham C57BL/10ScNJ (TLR-4 KO) group*. C57BL/10ScNJ (TLR-4 KO) mice were subjected to the surgical procedures, as reported above, except that the impact was not given (n = 30). As describe below mice (n = 10 from each group for each parameters) were killed at 24 h after TBI in order to evaluate the various parameter. In a separate set of experiments other 10 animals for each group were observed after TBI in order to evaluate the behavioral testing.

### Behavioral testing

TBI mice display motor and cognitive deficits. Thus, the present behavioral tests involved analyses of motor asymmetry (Elevated Biased Swing Test and Rotarod test). Training for the rotarod test was initiated at 1 week, respectively, before the CCI injury, whereas no training was required for EBST. The retard treadmill (Accuscan, Inc., Columbus, OH, USA) provided a motor balance and coordination assessment. Data were generated by averaging the scores (total time spent on treadmill divided by 5 trials) for each animal during training and testing days. Each animal was placed in a neutral position on a cylinder (3 cm and 1 cm diameter for rats and mice, respectively) then the rod was rotated with the speed accelerated linearly from 0 rpm to 24 rpm within 60 s, and the time spent on the retard was recorded automatically. The maximum score given to an animal was fixed to 60. For training, animals were given 5 trials each day and declared having reached the criterion when they scored 60 in 3 consecutive trials. For testing, animals were given 3 trials and the average score on these 3 trials was used as the individual rotorod score. The EBST provided a motor asymmetry parameter and involved handling the animal by its tail and recording the direction of the biased body swings. The EBST consisted of 20 trials with the number of swings ipsilateral and contralateral to the injured hemisphere recorded and expressed in percentage to determine the biased swing activity.

### Quantification of infarct volume

The mice were anesthetized with ketamine and decapitated. Their brains were carefully removed. The brains were cut into 5 coronal slices of 2 mm thickness. Slices were incubated in 2% solution of 2,3,5-triphenyltetrazolium chloride (TTC) at 37°C for 30 min and immersion fixed in 10% buffered formalin solution. TTC stains the viable brain tissue red, while infracted tissue remains unstained [16], [17]. For quantification of infracted area and volumes, the brain slices were photographed using a digital camera (Canon 4x, Canon Inc, China) and then Image analysis was performed on a personal computer with an image analysis software program (using an ImageJ for windows) [18]. To compensate the effect of brain edema, the corrected infarct area equals left hemisphere area minus (right hemisphere area minus infarct area) [19]. The corrected total infarct volume was calculated by summing the infarct area in each slice and multiplying it by slice thickness (2 mm).

### rCBF determinations

All rCBF mesurements were conducted with mice anesthetized with ketamine, using laser Doppler flowmetry PF-5010 (Periflux system, Jarfalla, Sweden) with relative flow values expressed as perfusion units [55]. For the mesurements mice were fixed in a stereotaxic apparatus, with the probe placed at the level of the dura directly above a small skull opening. Using a micromanipulator, two probes (probe 411, 0.45 mm in diameter) were positioned to cortical coordinates of 1.3 mm posterior to the bregma and 2.8 mm to each side of midline on the intact skull, being careful to avoid pial vessels after reflection of the skin overlying the calvarium. Because mouse skull and subarachnoid space are very thin, transcranial measurements of rCBF are consistent with craniectomy measurements [56]. The rCBF of both hemispheres were continuously measured for 15 minutes and averaged for each determination. All rCBF data was continuously stored in a computer and analyzed using the Perimed data acquisition and analysis system.

### Light microscopy

Tissue segments containing the lesion (1 cm on each side of the lesion) were paraffin embedded and cut into 5-lm-thick sections. Tissue sections were deparaffinized with xylene stained with Haematoxylin/Eosin (H&E) and studied using light microscopy (Dialux 22 Leitz). The segments of each brain contained the lesion (1 cm on each side of the lesion), were evaluated by an experienced histopathologist. Damaged neurons were counted and the histopathologic changes of the gray matter were scored on a 6-point scale [57]: 0, no lesion observed, 1, gray matter contained 1–5 eosinophilic neurons; 2, gray matter contained 5–10 eosinophilic neurons; 3, gray matter contained more than 10 eosinophilic neurons; 4, small infarction (less than one third of the gray matter area); 5, moderate infarction; (one third to one half of the gray matter area); 6, large infarction (more than half of the gray matter area). The scores from all the sections from each brain were averaged to give a final score for individual mice. All the histological studies were performed in a blinded fashion.

### Staining of mast cells and reticular fibre saturated with argentic

Brain sections were cut 5 μm thick and stained with 0.25% Toluidine blue, pH 2.5, for 45 min at room temperature. The sections were then dehydrated and mounted in xylene-based medium for viewing. Three non-sequential sections were chosen from one random block from each brain for examination. All sections were evaluated at 200x, while some sections were photographed at 400x using a Nikon inverted microscope.

### Immunohistochemical localization of GFAP, Chymase, Tryptase, IL-1β, iNOS, Nitrotyrosine, Poly-ADP-ribosyl polymerase (PARP), Bax and Bcl2

At the end of experiment, tissues were fixed in PBS-buffered formaldehyde and 8 µm sections were prepared from paraffin embedded tissues. After deparaffinization, endogenous peroxidase was quenched with 0.30% (v/v) hydrogen peroxide in 60% (v/v) methanol for 30 min. The sections were permeabilized with 0.1% (w/v) Triton X-100 in PBS for 20 min. Non-specific adsorption was minimized by incubating the section in 2% (v/v) normal goat serum in PBS for 20 min. Endogenous biotin or avidin binding sites were blocked by sequential incubation for 15 min with biotin and avidin, respectively. Sections were incubated overnight with (1) anti-nitrotyrosine rabbit polyclonal antibody (1∶1000 in PBS, v/v, Santa Cruz Biotechnology) or (2) anti-poly (ADP)-ribose (PAR) antibody (1∶500 in PBS, v/v, Santa Cruz Biotechnology) or (3) anti-chymase (purified human skin chymase) polyclonal antibody (1∶1000 in PBS, v/v, Santa Cruz Biotechnology) or (4) anti-tryptase (mast cell tryptase isolated from human lung) polyclonal antibody (1∶1000 in PBS, v/v, Santa Cruz Biotechnology) or (5) anti-iNOS (1∶1000 in PBS, v/v, DBA, Milan, Italy) or (6) anti-IL1β mouse monoclonal antibody (mouse anti bovine 1∶100, v/v, Serotec) or (7) anti-GFAP mouse monoclonal antibody (1∶100, v/v Santa Cruz Biotechnology) or (8) anti-bax (1∶500 in PBS, v/v, Santa Cruz Biotechnology) or (9) anti-Bcl2 (1∶500 in PBS, v/v Santa Cruz Biotechnology). Sections were washed and incubated with secondary antibody. Specific labeling was detected with a biotin-conjugated goat anti-rabbit IgG and avidin – biotin peroxidase complex (Vector Lab. Inc., Burlingame, CA). To verify the binding specificity for Nitrotyrosine, PAR, Chymase, Tryptase, iNOS, IL-1 β, GFAP and apoptosis (Bax, Bcl2) some sections were also incubated with only the primary antibody (no secondary) or with only the secondary antibody (no primary). Immunocytochemistry photographs (n = 5 photos from each samples collected from all mice in each experimental group) were assessed by densitometry as previously described [21], [22] by densitometry using Optilab Graftek software on a Macintosh personal computer.

### Western blot analysis for GFAP, P-JNK, IκB-α, NF-κB, Bax and Bcl2

Western blot was performed in the traumatic penumbra area from the ipsilateral injured brain and a similar area from the control and/or contralateral tissues using antibodies (Invitrogen Corporation, Carlsbad, CA, USA), and β-actin. Cytosolic and nuclear extracts were prepared as previously described [23] with slight modifications. Protein concentrations were determined using protein assay dye from Bio-Rad Laboratories (Hercules, CA, USA). The levels of GFAP, Bax, Bcl2, P-JNK, and IκB-α were quantified in cytosolic fraction and NF-κB was quantified in nuclear fraction from brain tissue collected after TBI at 24 h. The filters were blocked with 1xPBS, 5% (w/v) non fat dried milk (PM) for 40 min at room temperature and subsequently probed with specific Abs anti-Bax (1∶1000; Santa Cruz Biotechnology) or anti-Bcl2 (1∶500; Santa Cruz Biotechnology) or anti-phospho-JNK (Thr183/Tyr185; 1∶1000; Cell Signaling) or anti-GFAP (1∶2000; Santa Cruz Biotechnology) or anti-IκB-α (1∶1000; Santa Cruz Biotechnology) or anti-NF-κB p65 (1∶1000; Santa Cruz Biotechnology) in 1xPBS, 5% w/v non fat dried milk, 0.1% Tween-20 (PMT) at 4°C, overnight. Membranes were incubated with peroxidase-conjugated bovine anti-mouse IgG secondary antibody or peroxidase-conjugated goat anti-rabbit IgG (1∶2000, Jackson ImmunoResearch, West Grove, PA) for 1 h at room temperature. To ascertain that blots were loaded with equal amounts of protein lysates, they were also incubated in the presence of the antibody against β-actin protein (1∶5000 Sigma – Aldrich Corp.). The relative expression of the protein bands was quantified by densitometric scanning of the X-ray films with GS-700 Imaging Densitometer (GS-700, Bio-Rad Laboratories, Milan, Italy) and a computer program (Molecular Analyst, IBM), and standardized for densitometric analysis to β-actin levels.

### Materials

Unless otherwise stated, all compounds were obtained from Sigma-Aldrich Company Ltd. (Milan, Italy). All stock solutions were prepared in non-pyrogenic saline (0.9% NaCl; Baxter, Italy) or 10% DMSO.

### Statistical evaluation

All values in the figures and text are expressed as mean ± standard error of the mean (SEM) of N observations. For the *in vivo* studies N represents the number of animals studied. For comparisons among multiple groups, one-way analysis of variance (ANOVA), followed by post-hoc (Bonferroni) test, was used to determine significant differences. Differences between 2 groups were tested using the Student's t-test. In the experiments involving histology, the figures shown are representative of at least three experiments performed on different experimental days. Statistical significance was set at P<0.05.

## Supporting Information

Figure S1 **Colocalization of GFAP/Bax and GFAP/Bcl-2 after TBI.** Cells were double stained with antibodies against GFAP (green; a, b, c, d), Bax (red; c1, d1) and Bcl-2 (red; a1, b1). The yellow spots indicate the co-localizations (a, c2, d2). Brain sections revealed increased astrogliosis (GFAP+ cells) in TLR4WT (panel a, c). Slight GFAP immunoreactivity was present in TLR4KO (panel b, d). Bcl-2 expression was reduced by TBI in TLR4WT, while Bcl-2 immunoreactivity was yet present in brain cells from TLRKO. Reported images are representative of triplicate experiments. All images were digitalized at a resolution of 8 bits into an array of 2048×2048 pixels.
(TIF)

Figure S2 **Co-localization of Iba1/Bax and Iba1/Bcl-2 after TBI.** Cells were double stained with antibodies against Iba1 (green; a, b, c, d) and Bax (red; c1, d1) and Bcl-2 (red; a1, b1). Microglial cells (Iba1-positive cells) expressed Bax and Bcl-2 as shown in panel c2, d2, and a2, b2. All images were digitalized at a resolution of 8 bits into an array of 2048×2048 pixels.
(TIF)

Figure S3 **Colocalization of GFAP/NF-κB and GFAP/pJNK after TBI.** Cells were double stained with antibodies against GFAP (red; a1–d1), NF-κB (green; a and b) and pJNK (green; c and d). The yellow spots indicate the co-localizations of GFAP/NF-κB (a2–b2), and GFAP/pJNK (c2, d2). Brain sections revealed increased astrogliosis (GFAP+ cells) in TLR4WT (panel a1and c1). Slight GFAP immunoreactivity was present in TLR4KO (panel b1–d1). Reported images are representative of triplicate experiments. All images were digitalized at a resolution of 8 bits into an array of 2048×2048 pixels.
(TIF)

Figure S4 **Effect of absence of TLR4 on expression of NF-κB and pJNK in reactive micorglia (Iba1+ cells) after TBI.** Cells were double stained with antibodies against Iba-1(red; a1–d1), NF-κB (green; a and b) and pJNK (green; c and d). The yellow spots indicate the co-localizations of Iba-1/NF-κB (a2–b2), and Iba-1/pJNK (c2, d2). Iba1+ cells were present in the brain tissues from TLRWT mice (panels a1, c1) as well as TLR4KO mice (panels b1, d1) following TBI. Co-labelling of Iba-1/NF-κB and Iba1/pJNK are shown in panels a2–d2 in all brain sections.
(TIF)

Summary Diagram S1 **In our experimental design we performed TBI respectively in WT and TLR4KO mice.** TBI produces primary and secondary injury. The secondary injury is characterized by free radical production, apoptotic protein release (BAX, Chymase and tryptase), and inflammatory waterfall; in this step the NF-kB is activated with production of inflammatory gene transcription (IL-1β, inOS and nitrotyrosine).
(TIF)
