# Supplementary figures and images for: Absence of TLR4 Reduces Neurovascular Unit and Secondary Inflammatory Process after Traumatic Brain Injury in Mice
Source: PLoS One. 2013 Mar 28;8(3):e57208. doi: 10.1371/journal.pone.0057208 (PMC3610903; doi:10.1371/journal.pone.0057208)

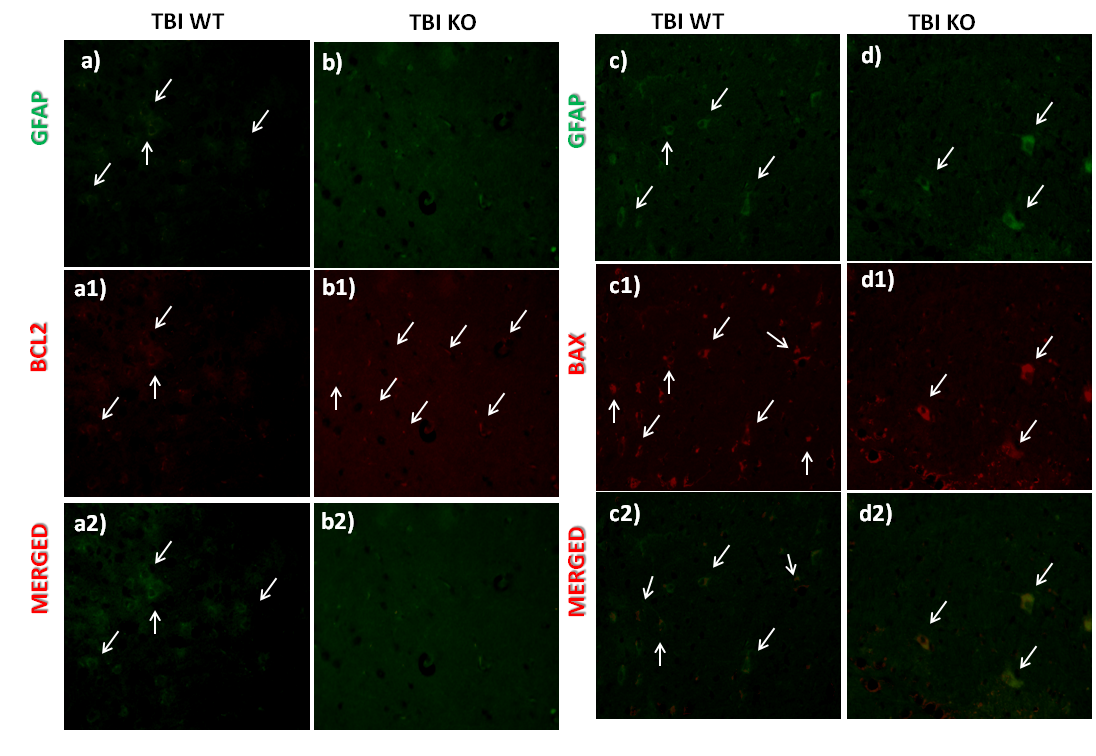

Supplement: Figure S1 — Colocalization of GFAP/Bax and GFAP/Bcl-2 after TBI. Cells were double stained with antibodies against GFAP (green; a, b, c, d), Bax (red; c1, d1) and Bcl-2 (red; a1, b1). The yellow spots indicate the co-localizations (a, c2, d2). Brain sections revealed increased astrogliosis (GFAP+ cells) in TLR4WT (panel a, c). Slight GFAP immunoreactivity was present in TLR4KO (panel b, d). Bcl-2 expression was reduced by TBI in TLR4WT, while Bcl-2 immunoreactivity was yet present in brain cells from TLRKO. Reported images are representative of triplicate experiments. All images were digitalized at a resolution of 8 bits into an array of 2048×2048 pixels. (TIF) [file pone.0057208.s001.tif]

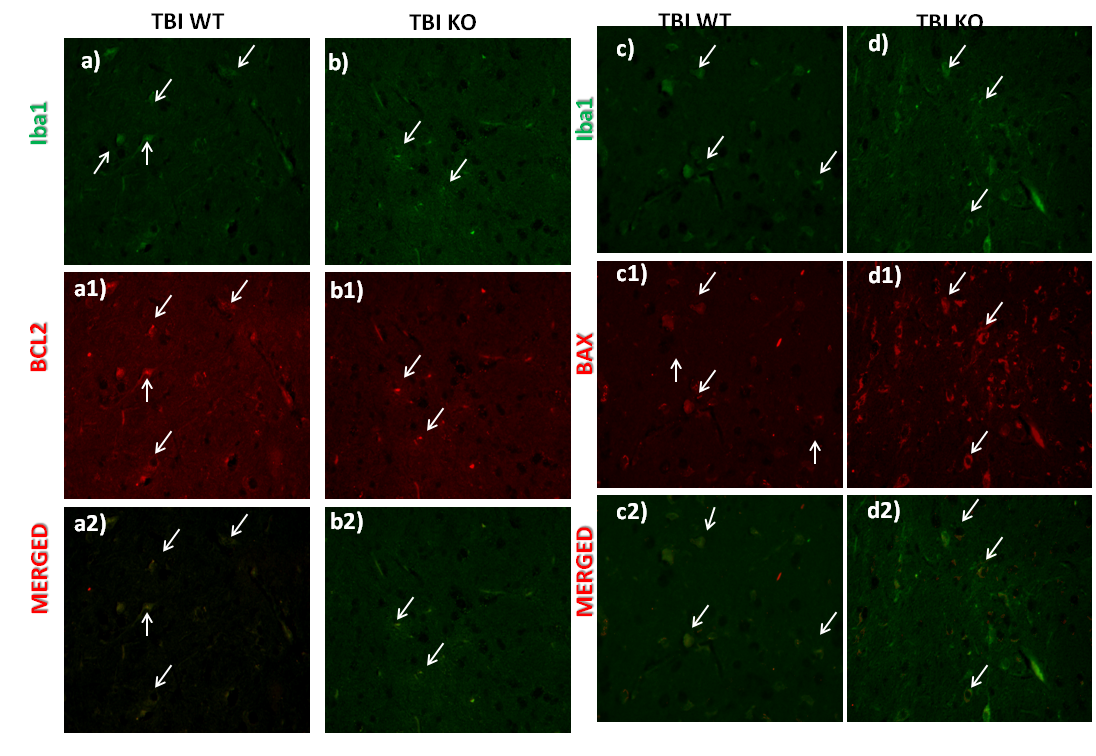

Supplement: Figure S2 — Co-localization of Iba1/Bax and Iba1/Bcl-2 after TBI. Cells were double stained with antibodies against Iba1 (green; a, b, c, d) and Bax (red; c1, d1) and Bcl-2 (red; a1, b1). Microglial cells (Iba1-positive cells) expressed Bax and Bcl-2 as shown in panel c2, d2, and a2, b2. All images were digitalized at a resolution of 8 bits into an array of 2048×2048 pixels. (TIF) [file pone.0057208.s002.tif]

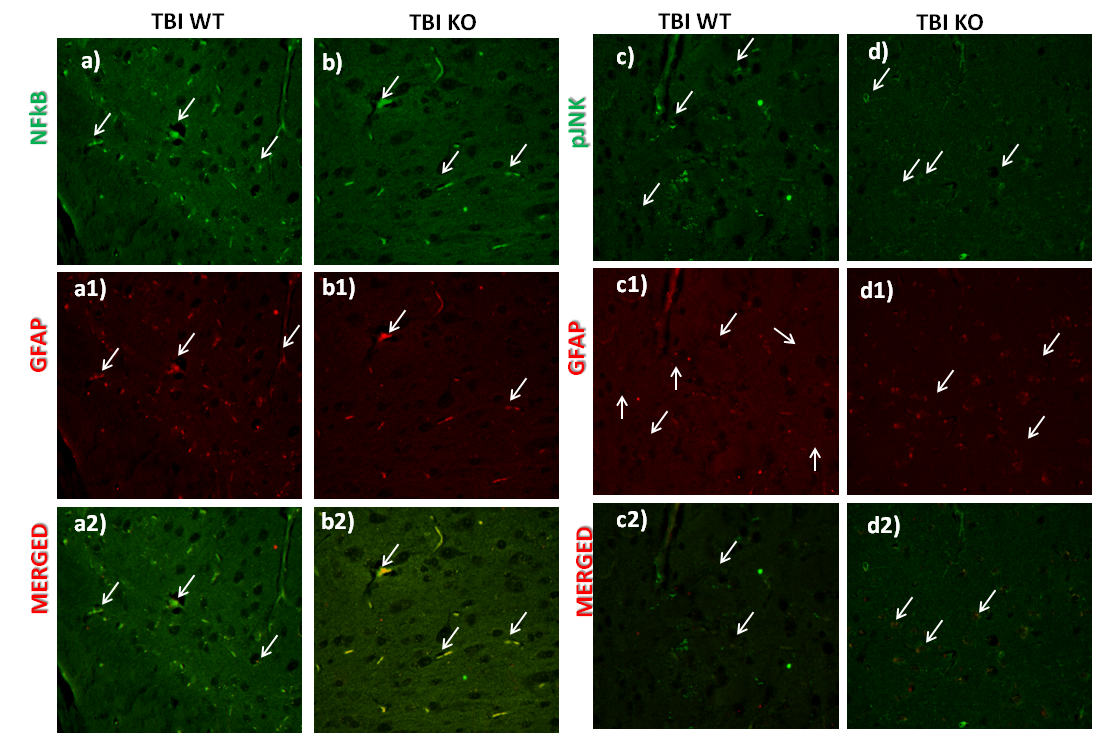

Supplement: Figure S3 — Colocalization of GFAP/NF-κB and GFAP/pJNK after TBI. Cells were double stained with antibodies against GFAP (red; a1–d1), NF-κB (green; a and b) and pJNK (green; c and d). The yellow spots indicate the co-localizations of GFAP/NF-κB (a2–b2), and GFAP/pJNK (c2, d2). Brain sections revealed increased astrogliosis (GFAP+ cells) in TLR4WT (panel a1and c1). Slight GFAP immunoreactivity was present in TLR4KO (panel b1–d1). Reported images are representative of triplicate experiments. All images were digitalized at a resolution of 8 bits into an array of 2048×2048 pixels. (TIF) [file pone.0057208.s003.tif]

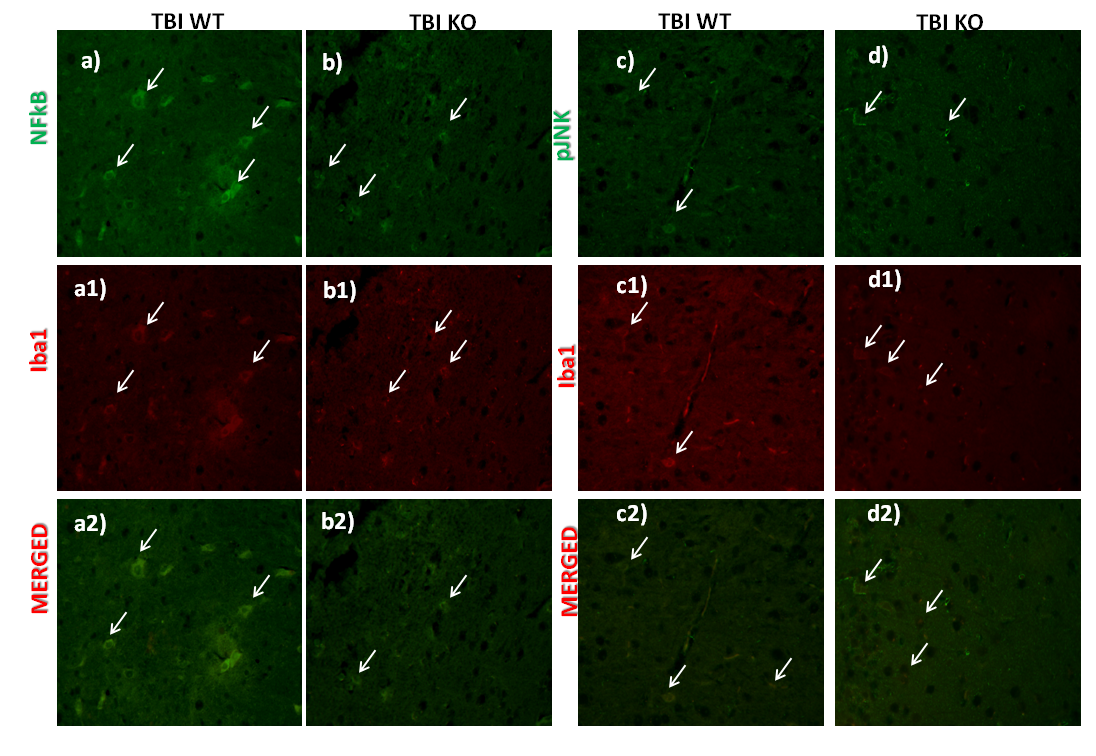

Supplement: Figure S4 — Effect of absence of TLR4 on expression of NF-κB and pJNK in reactive micorglia (Iba1+ cells) after TBI. Cells were double stained with antibodies against Iba-1(red; a1–d1), NF-κB (green; a and b) and pJNK (green; c and d). The yellow spots indicate the co-localizations of Iba-1/NF-κB (a2–b2), and Iba-1/pJNK (c2, d2). Iba1+ cells were present in the brain tissues from TLRWT mice (panels a1, c1) as well as TLR4KO mice (panels b1, d1) following TBI. Co-labelling of Iba-1/NF-κB and Iba1/pJNK are shown in panels a2–d2 in all brain sections. (TIF) [file pone.0057208.s004.tif]

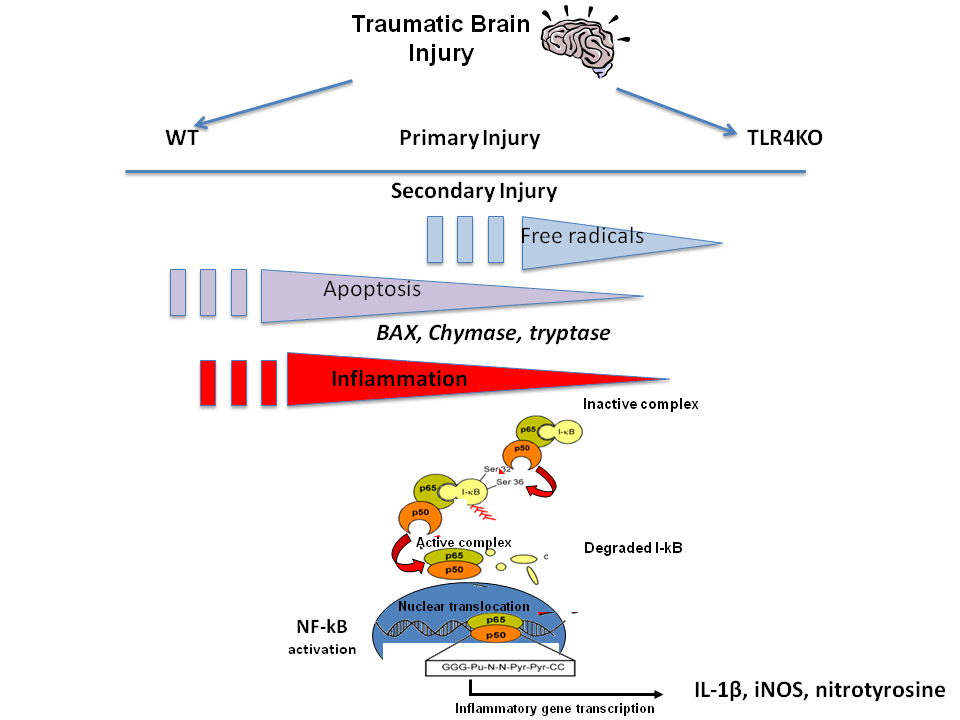

Supplement: Summary Diagram S1 — In our experimental design we performed TBI respectively in WT and TLR4KO mice. TBI produces primary and secondary injury. The secondary injury is characterized by free radical production, apoptotic protein release (BAX, Chymase and tryptase), and inflammatory waterfall; in this step the NF-kB is activated with production of inflammatory gene transcription (IL-1β, inOS and nitrotyrosine). (TIF) [file pone.0057208.s005.tif]
